# A Multi-Centre, Randomised, Double-Blind, Placebo-Controlled Phase III Clinical Trial Evaluating the Impact of BCG Re-Vaccination on the Incidence and Severity of SARS-CoV-2 Infections among Symptomatic Healthcare Professionals during the COVID-19 Pandemic in Poland—Evaluation of Antibody Concentrations

**DOI:** 10.3390/vaccines11010075

**Published:** 2022-12-29

**Authors:** Paweł Zapolnik, Wojciech Kmiecik, Anna Nowakowska, Łukasz Jerzy Krzych, Henryk Szymański, Lidia Stopyra, Teresa Jackowska, Dorota Darmochwał-Kolarz, Artur Mazur, Hanna Czajka

**Affiliations:** 1 College of Medical Sciences, University of Rzeszów, 35-315 Rzeszów, Poland; 2 St. Louis Provincial Specialist Children’s Hospital, 31-503 Kraków, Poland; 3 Medical Diagnostics Laboratory, Regional Sanitary-Epidemiological Station, College of Medical Sciences, University of Rzeszów, 35-315 Rzeszów, Poland; 4 Department of Anaesthesiology and Intensive Therapy, Faculty of Medical Sciences, Medical University of Silesia, 40-055 Katowice, Poland; 5 Saint Hedwig of Silesia Hospital, 55-100 Trzebnica, Poland; 6 Department of Infectious Diseases and Paediatrics, Stefan Żeromski Specialist Hospital, 31-913 Kraków, Poland; 7 Department of Paediatrics, Medical Centre of Postgraduate Education, 01-813 Warsaw, Poland

**Keywords:** COVID-19, BCG, vaccines, clinical trial, SARS-CoV-2, health care, antibodies

## Abstract

Tuberculosis (TB) was the predominant cause of death from a single infectious agent worldwide before the severe acute respiratory syndrome coronavirus type 2 (SARS-CoV-2) pandemic. Although TB vaccines have been successfully used for about 100 years, their full effect is still unknown. In previous studies, a reduced incidence and mortality from a coronavirus disease in TB-vaccinated populations were reported. In this article, we present the secondary analysis of a randomised controlled trial, reporting the results of a serological assessment evaluating the effect of the Bacillus Calmette–Guérin (BCG) vaccine on SARS-CoV-2. Participants—healthcare workers—were assessed 1–2 and 8 months after the second dose of the coronavirus disease 2019 (COVID-19) vaccine. We found no associations between antibody concentration, BCG revaccination, and additional characteristics, such as age, gender, or Body Mass Index. The effect of BCG vaccination on the immunological response against SARS-CoV-2 requires further research.

## 1. Introduction

Tuberculosis (TB) is a well-known infectious disease that, before the severe acute respiratory syndrome coronavirus 2 (SARS-CoV-2) pandemic, was the predominant cause of death from a single infectious agent worldwide [1]. Vaccination against the aetiological agent of tuberculosis, *Mycobacterium tuberculosis* (Bacillus Calmette–Guérin, BCG), was first used in 1921. The above vaccines are still recommended by the World Health Organization (WHO) in countries with an increased incidence of tuberculosis. In Poland, BCG prophylaxis has been administered since 1956, and from 2006 in the form of a single dose administered on the first day of life [2,3,4].

In studies conducted during the coronavirus disease pandemic 2019 (COVID-19) [5], as well as during previous observations of other infectious diseases [6,7,8,9,10,11], it was observed that there is a reduced incidence of disease and mortality from this particular disease in vaccinated populations. This is probably related to the effect of BCG’s stimulation of non-specific immunity as a form of ‘trained immunity’.

Since the onset of the COVID-19 pandemic, it has been hypothesised that the incidence of severe forms of the coronavirus disease has decreased in countries with long-standing TB prevention [12]. Observational and clinical studies have been initiated in many countries to confirm this hypothesis, including studies conducted on healthcare workers. The aim of our research is to evaluate the effect of revaccination with the BCG-10 vaccine (Biomed Lublin SA, Lublin, Poland) on the frequency and severity of SARS-CoV-2 infection among healthcare workers in Poland. The preliminary results of the study were presented in our previous paper [13]. In this article, we present the results of the antibody concentration assessment in the study subjects’ groups, and we relate them to individual characteristics such as age, gender, and Body Mass Index (BMI).

## 2. Materials and Methods

### 2.1. Methods

**Figure 1:**
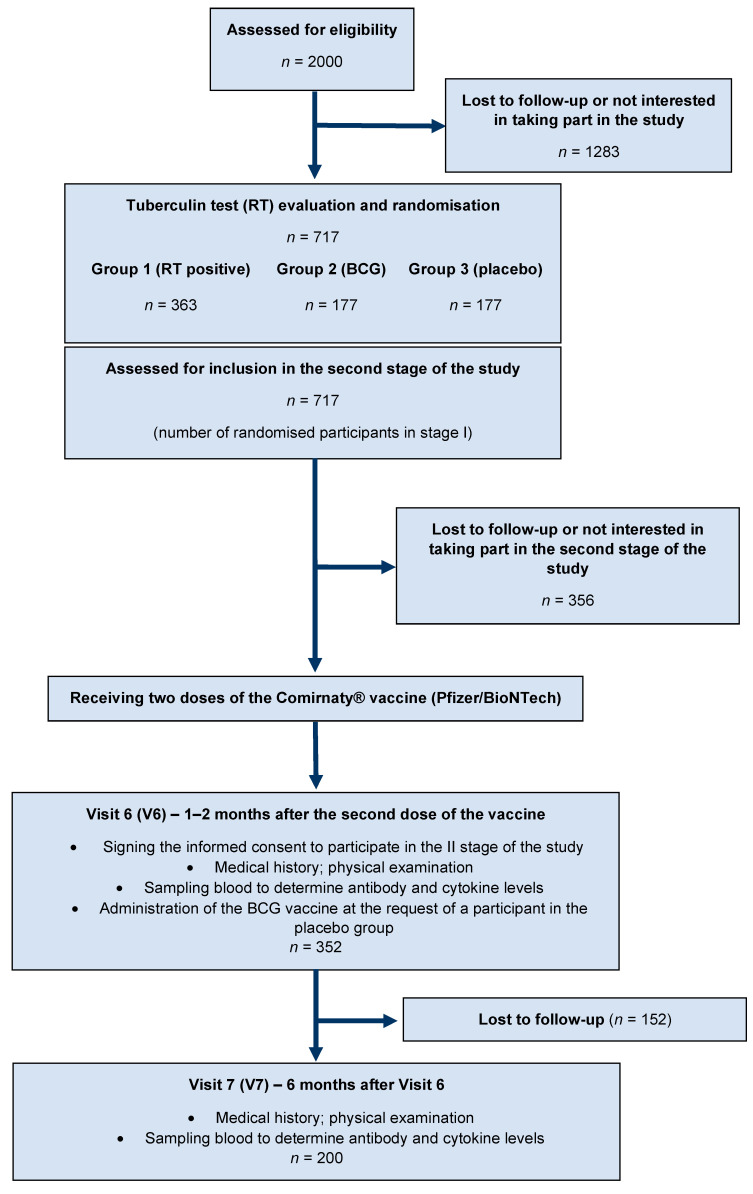
Flow diagram of the study.

Our study, conducted during the first phase in 6 centres in Poland, and in 4 centres during the second phase, was a clinical trial conducted on healthcare professionals (physicians, nurses, midwives, paramedics, laboratory diagnosticians, electroradiology technicians, physiotherapists, nutritionists, and orderlies). At the outset of the study, we assessed 2000 potential participants against the inclusion criteria described in more detail in our previous work [13]. Ultimately, 717 participants were included for randomisation, of whom 352 participants (visit V6) and 200 participants (visit V7) were assessed in the second stage of the study, as shown in Figure 1. The inclusion period of the study was from July to December 2020. The study received a positive opinion from the Bioethics Committee of the University of Rzeszów (No. 01/05/2020 of 06/05/2020). Before inclusion in the study, participants signed an informed consent. Study drop-out was based on the participant’s decision to terminate participation in the study. The study was registered at ClinicalTrials.gov (NCT04648800) [14].

### 2.2. Intervention

In the first stage of the study, BCG immunisation was assessed via the RT23 tuberculin test. After 72 h, the evaluation was performed, and patients with negative test results were randomised in a 1:1 ratio. Half of the participants received the BCG-10 vaccine (BIOMED Lublin SA, Lublin, Poland), and half received a placebo. During the 3-month evaluation, a nasopharyngeal swab was performed if symptoms suggestive of COVID-19 occurred. In the second phase of the study, after the introduction of widespread vaccination against COVID-19, patients were assessed 1–2 months after receiving the second dose of the Comirnaty® vaccine (Pfizer/BioNTech, New York, NY, USA/Mainz, Germany). In this article, we focus on the serological assessment of the participants; we described the broader methodology of the study in a previous paper [13].

#### 2.2.1. Outcome Measures

The primary outcome of the study in a serological context was the determination of anti-SARS-CoV-2 antibody concentrations in the three patient groups studied (RT23+, BCG, placebo). A detailed description of the method is provided in Section 2.3. Secondary outcomes included determining the relationship in antibody concentrations with age, sex, and Body Mass Index.

#### 2.2.2. Allocation and Blinding

We used the electronic Case Report Form (e-CRF) system to perform randomisation. The medical staff of the study were divided into unblinded and blinded. Blinded records were kept separately and secured from access by other study team members. At visit two (V2), the results of the RT23 tuberculin test were read, and participants with a test > 6 mm remained in group I (first). The test result was entered into the e-CRF, and a negative result of 0–5 mm determined automatic randomisation involving random allocation (1:1) to group II (second), receiving the BCG vaccine, or to group III (third), receiving placebo. Information on allocation to a specific group (II or III) was kept in written records. No information on the effect of randomisation was available in the e-CRF programme. The allocation sequence was also secured. Both vaccine and placebo were administered intradermally in a volume of 0.1 mL. The study was blinded to participants, investigators, and data-collection and statistical-analysis personnel until the study was unblinded at the end of phase I (V5).

### 2.3. Serological Tests

Blood for serological testing of individuals exposed to SARS-CoV-2 infection or after vaccination was assessed by quantitative enzyme-linked immunosorbent assay (ELISA) with a determination of the level of immunoglobulin G-class (IgG) antibodies against the S1 antigen of SARS-CoV-2 virus. The assay was performed on an Analyser I-2P immunoassay analyser from EUROIMMUN, using the commercial Anti-SARS-CoV-2 QuantiVac ELISA IgG assay from EUROIMMUN Medizinische Labordiagnostika AG, Lübeck, Germany. The reaction wells were coated with the S1 domain of the SARS-CoV-2 protein S (spike protein) expressed by recombination in the human cell line HEK 293. In the first step of IgG antibody identification, patient sera were diluted in the appropriate buffer in a ratio of 1:101. If the test serum extinction exceeded 120 RU/mL (i.e., the highest reading on the calibration curve), further 10-fold dilutions, i.e., 1:1010, 1:10,100, etc., were performed. All dilutions and further analysis steps were performed automatically (Analyser I-2P). During the course of the individual analyses, the validity of the assays performed was assessed based on the extinction readings of the positive control and negative control. The calibration curve for the assay system was plotted as a point-to-point plot for 6 calibrators with values of 1 RU/mL, 10 RU/mL, 20 RU/mL, 40 RU/mL, 80 RU/mL, and 120 RU/mL (relative units/mL). For the test used, the test manufacturer reported a limit of detection for the analyte of 1.20 RU/mL, and for the blank (background) sample, of 0.86 RU/mL. For the evaluation of the extinction of the serum samples, the following criteria were adopted for the analyses performed: <8 RU/mL—Negative result; ≥8 to <11 RU/mL—Borderline result; ≥11 RU/mL—Positive result. A positive result meant a finding of anti-SARSCoV-2 IgG-class antibodies; a negative result meant no finding of anti-SARS-CoV-2 IgG antibodies; a borderline result meant an inconclusive seroconversion assessment. In addition to the relative units/mL read, parallel analysis results were reported in BAU/mL (binding antibody units/mL), which has been recognised as the standard for international units (First WHO International Standard for anti-SARS-CoV-2 immunoglobulin. 2021. https://www.who.int/groups/expert-committee-on-biological-standardization (accessed on 12 April 2022)). The following interpretation of the analyses performed was adopted to assess the extinction of serum samples in the new units: <25.60 BAU/mL—Negative result; ≥25.60 to <35.20 BAU/mL—Borderline result; ≥35.20 BAU/mL—Positive result.

### 2.4. Statistical Analysis

Statistical evaluation was performed using MedCalc v17.7. Software (MedCalc Software Ltd., Ostend, Belgium). Quantitative variables were presented in the following forms: arithmetic mean and standard deviation (variables with a normal distribution), or median and interquartile range (variables with non-normal distribution). Differences between groups for quantitative variables were assessed by either Student’s *t*-test or analysis of variance (independent samples, variables with normal distribution), or Mann–Whitney U or Kruskal–Wallis (independent samples, variables with skewed distribution). To assess the significance of differences for quantitative dependent variables, the Student’s *t*-test for dependent samples or the non-parametric equivalent of the analysis of variance, the Wilcoxon paired-rank order test, or the Friedman test (depending on the number of groups and the nature of the distribution) was used. The Shapiro–Wilk test was used to assess the nature of the distribution. Qualitative variables will be presented as absolute values and percentages. We used the chi-square test or Fisher’s exact test for unrelated qualitative variables. Finally, the significance criterion was taken at *p* < 0.05. The statistical analysis was a per-protocol analysis.

## 3. Results

For the final assessment of antibody levels, 352 subjects were included at visit V6, and 200 at visit V7, of the initial 717 randomised study participants. The diagram of the second stage of the study (visits V6 and V7) is presented in Figure 1. The chart of the first stage of the research and its results are available in our previous article [13].

Antibody concentrations in the three study groups measured at visit V6 were not significantly different: *p* = 0.89 (V6), *p* = 0.32 (V7). The results of the serological determinations, along with the standard deviation, are presented in Table 1.

The correlation of antibody concentration with the age structure of the study population also showed no significance: *p* = 0.13 (V6), *p* = 0.49 (V7), (Table 2).

Another parameter analysed in our study was the relationship of antibody concentration to Body Mass Index (BMI). In this context, statistical significance could not be proved either: *p* = 0.37 (V6), *p* = 0.31 (V7), (Table 3).

In the following tables (Table 4 and Table 5), we present antibody concentrations along with standard deviations in the gender and age structure in relation to the BMI range.

## 4. Discussion

After observing a reduced incidence of COVID-19 in countries continuing universal tuberculosis prophylaxis, various studies were initiated to confirm this hypothesis. The results of studies from different centres are not conclusive, nor is the methodology of the analyses performed.

Weng et al. [5] studied a cohort of 120 adult patients with COVID-19 in March and April 2020. Eighty-two patients (68.3%) were vaccinated with BCG, and this group had a lower risk of hospitalisation (*p* = 0.019). However, the authors did not assess anti-SARS-CoV-2 antibody levels, so we cannot directly compare our results with this outcome. In addition, in the above study, people who had never been vaccinated with BCG were present, which is a significant difference.

Amirlak et al. [15] conducted a study in which 280 healthcare workers were offered BCG immunisation. All 280 had been previously vaccinated against tuberculosis; of this group, 71 received an additional dose, and 209 did not. During a follow-up of more than three months, the authors recorded 18 cases of SARS-CoV-2 infection among hospital staff. All cases were in the group that did not receive an additional dose of the BCG vaccine. The authors showed statistical significance, but, as in the work of Weng et al. [15], antibody concentrations were not assessed here [15]. Despite the similar age and the population being previously vaccinated with BCG, we cannot compare the results of our serological assessment. 

Khanum et al. [16] conducted an observational study on adult participants with a positive polymerase chain reaction (PCR) test for SARS-CoV-2. The study evaluated 103 patients with COVID-19, of whom 64 participants had previously received BCG vaccination (scar-based assessment), and 39 had not been vaccinated against TB. The authors did not demonstrate statistical significance in terms of the severity of the COVID-19 disease in the groups compared. Still, the previously vaccinated group showed significantly lower mortality than previously unvaccinated patients [16]. We did not evaluate COVID-19 mortality in our study. The different nature of the evaluation—observation of patients with the disease rather than a randomised clinical trial with vaccine administration—also differs from our work in the cited study. Consequently, it is difficult to predict what such a finding would look like in our study population.

In another multicentre observational study, Torun et al. [17] analysed 465 healthcare workers with SARS-CoV-2 infection for a history of contact and exposure to *Mycobacterium tuberculosis*. The authors showed that the hospitalised study participants had a history of direct contact with tuberculosis patients. In addition, a higher number of working hours in the hospitalised participants was proven. On the other hand, only one person in the study population died due to COVID-19, so the overall mortality rate was low. Such results can probably be linked to the process of ‘trained immunity’, which is influenced by BCG vaccination and perhaps also by direct contact with TB. On the one hand, ‘trained immunity’ enhances the anti-viral response, but, on the other hand, it may stimulate a Th1/Th17-type inflammatory response and generate severe COVID-19 disease symptoms [18]. The results of the above-cited study could correspond to such a process, but the limitation here is that the study is observational in nature rather than a randomised clinical trial. The authors did not analyse the concentration of antibodies in the participants, either. For these reasons, it is also difficult to directly compare the results of Torun et al. with our study.

The results of Rivas et al. [19], who analysed more than 6000 healthcare workers for BCG vaccination, are different. They also determined IgG-class anti-SARS-CoV-2 antibody titres. A total of 29.6% participants were vaccinated, while 68.9% were not. The reporting of COVID-19 disease symptoms and seroprevalence based on the determination of anti-SARS-CoV-2 antibody titres was significantly lower in the BCG-vaccinated group of medical workers compared to the unvaccinated group. The authors additionally analysed the study participants regarding vaccination against *Neisseria meningitidis*, *Streptococcus pneumoniae* and influenza, but did not find any comparable results [19]. In the above study, some characteristics are similar to ours, such as the participants’ mean age and profession. However, this is not a randomised clinical trial but a retrospective clinical study. In addition, given the different vaccinability of the population, it can be concluded that our results are entitled to differ from those of Rivas et al.

The nature of the study by Tsilika et al. [20] is much more similar to ours compared to the previously cited work. The authors conducted a randomised, double-blind clinical trial on 516 elderly participants (median age 68 years). Ultimately, 301 participants received BCG or a placebo, and the follow-up period was six months. Re-vaccination with BCG reduced the risk of COVID-19 to 68%. In addition, the authors collected blood from 300 participants 3 months after vaccination to assess SARS-CoV-2 antibody levels to detect those who had undergone asymptomatic infection. A positive serological result was obtained in 1.3% (2/153) of the subjects in the placebo group and in 4.7% (7/148) in the BCG group, which was not statistically significant (*p* = 0.099). A randomised clinical trial was used in the above study, as in ours. The study participants were also previously vaccinated with BCG due to Greece’s vaccination policy. They were not healthcare workers, but, as in our case, no statistical significance was shown. The authors did not analyse other characteristics, such as age or gender, in relation to antibody concentrations, so it is difficult to compare our overall results with the paper mentioned above.

Uysal et al. [21] conducted a study to evaluate the serological response after COVID-19 vaccination in healthcare workers, analysing factors such as age, gender, smoking habits, and BMI. The authors only showed a statistically significant difference for smoking, with higher antibody titres obtained in non-smokers. However, no significance was shown in the other parameters, as in our study. Nonetheless, the above study did not assess the effect of BCG vaccination on COVID-19 or serological response.

A study relatively similar to ours was performed by Dos Anjos et al. [22], who conducted a single-centre clinical trial of healthcare workers with revaccination with the BCG Moscow strain. Although the authors presented a reduction in the incidence of COVID-19 in the vaccinated group in their results, they did not reach any statistical significance. However, a placebo was not used in this study. An additional significant difference from our trial was that the participants included in the analysis worked at least eight hours per week in direct contact with patients suspected of COVID-19. Therefore, our results cannot be directly compared.

A multicentre clinical trial with placebo control was conducted by Jalalizadeh et al. [23] on a group of 378 adult patients, not medical professionals but people recovering from the COVID-19 disease. The participants in the BCG group had a greater return of smell and taste within six weeks of follow-up compared to the placebo group. In addition, a reduced risk of ageusia in the following weeks was shown among those vaccinated with BCG. However, these data do not correlate with ours due to the different participant profiles and the nature of the study. In a similar study, Dionato et al. [24] assessed the safety of BCG revaccination in patients in recovery from COVID-19, finding no adverse effects, and additionally showing a faster return of the sense of smell in the vaccinated group.

Moorlag et al. [25] conducted a randomised trial in elderly patients (>60 years of age) with BCG and a placebo. They focused on assessing the total incidence of respiratory-tract infections requiring medical intervention. BCG did not affect the incidence of infections, including COVID-19, but it is worth noting that, in patients diagnosed with the SARS-CoV-2 infection, antibody titres were higher if the participant had previously received BCG. This result differs from our study, but this was not the authors’ primary aim, unlike in our team. The humoral response and antibody production after COVID-19 infection and the impact of BCG require further study.

Another interesting study was conducted by Ten Doesschate et al. [26]. This was structurally similar to ours: a multicentre clinical trial of healthcare workers randomised 1:1 with BCG and placebo. However, the main difference was in the characteristics assessed; the authors analysed the number of days of absenteeism from work without showing statistical significance. In our study, we did not consider such a parameter at all.

A study to evaluate the efficacy of BCG on COVID-19 in a group of adult patients with type I diabetes was conducted by Faustman et al. [27]. In the BCG-vaccinated group, participants had a lower incidence of COVID-19 and infectious disease symptoms, and lower titres of IgG antibodies to SARS-CoV-2 than the placebo group. However, the promising results of the study only apply to one population, and, in addition, one with a high-risk: Type I diabetes. In our trial, we did not analyse participants by additional comorbidities, but by their profession, age, gender, or BMI [13].

The most similar study to ours was the Australian population trial conducted by Pittet et al. [28]. This study is also a multicentre phase III clinical trial in which participants —healthcare workers—were randomised in a 1:1 ratio, with one group receiving the BCG vaccine produced in Denmark together with the influenza vaccine, and the other group receiving the influenza vaccine alone. Participants were assessed for a total of 12 months for the frequency and severity of the SARS-CoV-2 virus infection, and, as in our study, blood samples were taken from them to assess antibody levels. Unfortunately, the results of this study, which could be a precious reference to compare to our results, have not been published so far.

In the discussion on the current state of knowledge, it is also worth having a look at the comment by Netea et al. [29]. They highlighted the incomplete status of knowledge on the effect of BCG vaccination on the course of the COVID-19 disease and the need to publish the results of randomised clinical trials. They also mentioned that some studies suggest the efficacy of the BCG effect when the vaccine was administered after birth instead of as revaccination already during the pandemic. 

Our study was not without limitations. Primary among these was the lack of baseline antibody concentrations upon vaccination for SARS-CoV-2, which, however, was not the original project assumption. Perhaps because of this, it was impossible to demonstrate relevance in the context of vaccination and the period since the second dose of the vaccine. Another limitation of our study was the lack of a control group, never vaccinated. In the study population, participants received at least two doses of the BCG vaccine, as BCG prophylaxis has been mandatory in Poland since 1955. This fact probably hindered the evaluation and contributed to this result. It is also worth mentioning that the evaluation of antibody concentrations we carried out was of a scientific nature, with no intention of making specific recommendations in practice.

Due to the lack of published results from analogous studies, it is challenging to conclude conclusively whether BCG revaccination can help control the SARS-CoV-2 coronavirus pandemic or reduce the severity of COVID-19. It is also difficult to compare our serological results with other studies, as their different methodologies do not allow complete translation of the results. Further research on the effect of BCG on the coronavirus disease is necessary, not least because of the possible use of this vaccine as an additional tool against a pandemic. This is especially relevant for developing countries, where the availability of SARS-CoV-2 vaccines, as well as storage capacity, is limited.

## 5. Conclusions

Knowledge of the BCG vaccine is over a century old, and the SARS-CoV-2 coronavirus pandemic is now more than two years away. Despite this, we still need to learn all the mechanisms of immune interactions in TB-vaccinated and non-vaccinated individuals after contact with SARS-CoV-2. Based on the observations and the first results of various studies, we can suspect that BCG influences the response against coronavirus, most likely through a process of trained immunity, which is an enhanced response to the infectious agent through innate immunity following previous BCG stimulation. Looking at the SARS-CoV-2 infection generally, it is worth noting that we do not know all the mechanisms by which the virus affects human cells. However, there are growing reports indicating possible mitochondrial dysfunction, both in terms of the respiratory chain and the destruction of the mitochondrial membrane [30,31,32]. It is also worth mentioning that research is currently being conducted on anti-COVID-19 agents other than vaccines or classical antiviral drugs. Nanoparticles (e.g., polymeric or peptide-based) can act as carriers or adjuvants in vaccines but also form antiviral surfaces or biosensors. Thus, nanotechnology can provide an additional tool in COVID-19 diagnosis and therapy [33].

Clearly, this area requires further study and in-depth knowledge. We know that the Polish population is relatively homogeneous in terms of vaccination against tuberculosis due to the national health policy over the years. Presumably, the lack of significance in our study may be due to this fact. In this enlightened context, conducting an analogous controlled clinical trial among health professionals in a more diverse population would seem appropriate. It is also worth mentioning that, in our study, we did not analyse additional factors that may influence the immune response process, such as co-morbidities, medications taken by the participants, or the type of diet of the participants. In our opinion, future studies should also pay attention to these aspects, so that the knowledge of the impact of BCG vaccination on COVID-19 is complete and perhaps opens the way to new diagnostic and therapeutic solutions.

## Figures and Tables

**Table 1:** Antibody concentrations in the three study groups.

**Groups of Patients**	**Number of Participants from V6**	**Mean from V6 [BAU/mL]**	**SD from V6 [BAU/mL]**	**Number of Participants from V7**	**Mean from V7 [BAU/mL]**	**SD from V7 [BAU/mL]**
Group 1 (RT23+) Group 2 (BCG) Group 3 (Placebo)	156	1277.39	1550.63	88	482.22	1432.77
	100	1344.88	1728.23	55	446.87	787.49
	96	1172.91	1099.58	57	336.93	304.54
**Total**	**352**	**1268.07**	**1495.21**	200	**431.09**	**1046.71**

Abbreviations: BAU—Binding antibody units; BCG—Bacillus Calmette–Guérin; SD—Standard deviation. Numbers in bold represent the total number of participants and serological totals.

**Table 2:** Antibody concentration according to the age of the study participants.

**Age**	**Number of Participants from V6**	**Mean from V6 [BAU/mL]**	**SD from V6 [BAU/mL]**	**Number of Participants from V7**	**Mean from V7 [BAU/mL]**	**SD from V7 [BAU/mL]**
<35 35–50 >50	92	1261.38	959.50	44	321.81	254.83
	126	1117.29	1348.51	71	337.95	571.73
	134	1414.44	1875.62	85	565.47	1502.68
**Total**	**352**	**1268.07**	**1495.21**	**200**	**431.09**	**1046.71**

Abbreviations: BAU—Binding antibody units; SD—Standard deviation. Numbers in bold represent the total number of participants and serological totals.

**Table 3:** Antibody concentration according to the BMI of the study participants.

**BMI**	**Number of Participants from V6**	**Mean from V6 [BAU/mL]**	**SD from V6 [BAU/mL]**	**Number of Participants from V7**	**Mean from V7 [BAU/mL]**	**SD from V7 [BAU/mL]**
<18.5 18.5–24.99 25–29.99	13.00	943.02	753.94	5.00	414.94	398.73
	196.00	1273.79	1336.41	102.00	283.27	375.96
	93.00	1292.84	1910.24	60.00	649.88	1765.92
**Total**	**352**	**1268.07**	**1495.21**	**200**	**431.09**	**1046.71**

Abbreviations: BAU—Binding antibody units; BMI—Body Mass Index; SD—Standard deviation. Numbers in bold represent the total number of participants and serological totals.

**Table 4:** Antibody concentrations in the gender structure in relation to BMI.

**BMI/Gender**	**Number of Participants from V6**	**Mean from V6 [BAU/mL]**	**SD from V6 [BAU/mL]**	**Number of Participants from V7**	**Mean from V7 [BAU/mL]**	**SD from V7 [BAU/mL]**
**<18.5**	**13.00**	**943.02**	**753.94**	**5.00**	**414.94**	**398.73**
Women	12.00	947.04	787.32	5.00	414.94	398.73
Men	1.00	894.78	-	-	-	-
**18.5–24.99**	**196.00**	**1273.79**	**1336.41**	**102.00**	**283.27**	**375.96**
Women	172.00	1318.35	1397.67	94.00	293.06	389.06
Men	24.00	954.42	704.98	8.00	168.24	109.09
**25–29.99**	**93.00**	**1292.84**	**1910.24**	**60.00**	**649.88**	**1765.92**
Women	67.00	1356.79	2152.80	46.00	774.45	2000.45
Men	26,00	1128.03	1074.22	14.00	240.60	255.80
**>30**	**50.00**	**1284.09**	**1373.70**	**33.00**	**492.65**	**659.00**
Women	37.00	1319.34	1522.36	24.00	524.64	744.98
Men	13.00	1183.78	859.10	9.00	407.33	360.91
**Total**	**352.00**	**1268.07**	**1495.21**	**200.00**	**431.09**	**1046.71**

Abbreviations: BAU—Binding antibody units; BMI—Body Mass Index; SD—Standard deviation. Numbers in bold represent the total number of participants and serological totals depending on the particular feature.

**Table 5:** Antibody concentrations in the age and gender structure in relation to BMI.

**Age/BMI/Gender**	**Number of Participants from V6**	Mean from V6 [BAU/mL]	SD from V6 [BAU/mL]	Number of Participants from V7	Mean from V7 [BAU/mL]	SD from V7 [BAU/mL]
**<35**	**92**	**1261.38**	**959.50**	**44**	**321.81**	**254.83**
**<18.5**	**9**	**1008.14**	**736.35**	**3**	**601.88**	**421.18**
Women	8	1022.31	785.88	3	601.88	421.18
Men	1	894.78	-	**-**	**-**	**-**
**18.5–24.99**	**69**	**1312.76**	**1021.79**	**31**	**305.68**	**244.79**
Women	57	1359.62	1067.24	27	317.84	257.14
Men	12	1090.16	768.17	4	223.66	125.92
**25–29.99**	**10**	**1175.19**	**575.39**	**7**	**263.55**	**197.79**
Women	6	885.99	448.30	4	234.27	203.88
Men	4	1608.99	489,98	3	302.60	225.87
**>30**	**4**	**1160.26**	**1237.03**	**3**	**344.32**	**261.23**
Women	1	400.00	-	-	-	-
Men	3	1413.67	1382.04	3	344.32	261.23
**35–50**	**126**	**1117.29**	**1348.51**	**71**	**337.95**	**571.73**
**<18.5**	**3**	**1012,11**	**949.11**	**1**	**232.17**	**-**
0	3	1012.11	949.11	1	232.17	-
**18.5–24.99**	**70**	**1029.84**	**915.13**	**37**	**212.02**	**154.34**
Women	62	1032.03	944.84	34	218.41	159.35
Men	8	1012.82	689.23	3	139.63	35.68
**25–29.99**	**30**	**1291.84**	**2258.60**	**16**	**539.74**	**1117.97**
Women	21	1380.08	2682.26	13	552.58	1235.48
Men	9	1085.95	661.59	3	484.12	458.15
**>30**	**23**	**1169.50**	**910.56**	**17**	**428.31**	**343.43**
Women	17	1143.50	955.37	12	397.72	313.33
Men	6	1243.15	847.70	5	501.75	438.45
**>50**	**134**	**1414.44**	**1875.62**	**85**	**565.47**	**1502.68**
**<18.5**	**1**	**149.71**	**-**	**1**	**36.89**	**-**
0	1	149.71	-	1	36.89	-
**18.5–24.99**	**57**	**1526.21**	**1943.78**	**34**	**340.36**	**585.33**
Women	53	1608.91	1991.09	33	349.70	591.83
Men	4	430.39	298.82	1	32.41	-
**25–29.99**	**53**	**1315.60**	**1884.29**	**37**	**770.60**	**2129.36**
Women	40	1415.18	2022.37	29	948.41	2382.63
Men	13	1009.18	1399.46	8	126.03	70.05
**>30**	**23**	**1420.23**	**1768.99**	**13**	**611.01**	**981.27**
Women	19	1525.05	1924.32	12	651.57	1013.46
Men	4	922.31	574.54	1	124.30	-
**Total**	**352**	**1268.07**	**1495.21**	**200**	**431.09**	**1046.71**

Abbreviations: BAU—Binding antibody units; BMI—Body Mass Index; SD—Standard deviation. Numbers in bold represent the total number of participants and serological totals depending on the particular feature.

## Data Availability

EudraCT No. 2020-002111-22.

## Abbreviations

BAU	Binding antibody units
BCG	Bacillus Calmette–Guérin
BMI	Body Mass Index
COVID-19	Coronavirus disease 2019
e-CRF	electronic Case Report Form
ELISA	Enzyme-linked immunosorbent assay
IgG	Immunoglobulin G
PCR	Polymerase chain reaction
RU	Relative units
SARS-CoV-2	Severe acute respiratory syndrome coronavirus 2
TB	Tuberculosis
Th1	T helper type 1
Th17	T helper type 17
WHO	World Health Organisation
